# Supplementary material for: Factors associated with risk of HIV-infection among pregnant women in Cameroon: Evidence from the 2016 national sentinel surveillance survey of HIV and syphilis
Source: PLoS One. 2019 Apr 12;14(4):e0208963. doi: 10.1371/journal.pone.0208963 (PMC6461244; doi:10.1371/journal.pone.0208963)
Supplement: S1 Table — (DOCX) [file pone.0208963.s001.docx]

| **1. Patient code:____________________________________________________**  **4. Date of patient visit (dd/mm/yyyy):_______/______/________**  **5. Age (in yearrs):___________________ 6. Residence : □** Urban **□** Rural | | | |
| --- | --- | --- | --- |
| **N° *Encercler la lettre qui correspond à la bonne réponse.*** | | | |
| **7. Highest level of school attended:**   1. *Never been in school 3. Secondary* 2. *Primary 4. Higher* 3. *Dont know* |  | **8. Marital status**  *1. single 2. Maried/*  *3. divorced/widower* |  |
| **9. Primary Occupation :**   1. *Not employed 5. Farmer* 2. *Housewife 6. Domestic help* 3. *Student 7. Laborer* 4. *Police/military 8 Other (specify)* ____________________________________ |  | **10. Total number of pregnancies, including this pregnancy:**  \|_____\|_____\| |  |
|  |  | **11. Total number of live births:**  \|____\|_____\| |  |
| **12. Former HIV status of the woman :**  1*. Positive 2. Negative*  *3. Never been tested* |  | **13 What is the first HIV test result (on site Lab )**  *1. Positive 2. Negative*  **14. Date of first HIV test :**  **________/________/________** |  |
| **13. Date of the former HIV sreening :**  **________/________/________** |  |  |  |
| **15. Was the HIV test accepted by the woman?**  *1. Yes 2. No* |  | **16. What is the second HIV test result (On site Lab):**  *1. Positive 2. Negative*  **17. Date of second HIV test :**  **________/________/________** |  |
| **18. Date the syphilis test :**  **_____/______/________**  **19. Result of the syphilis test:**  *1. Positive 2. Negative* |  | **20. HIV test result from the reference lab (To be fill by NACC)**  *1. Positive 2. Negative*  **21. Date of HIV test at the reference lab :**  **________/________/________** |  |

***ANC Data Collection Form***

1. **Region: _________________________________ 2. Health Facility______________________________**
